# Inclusion of planetary health and Sustainable Development Goals in nursing training

**DOI:** 10.1590/0034-7167-2024-0014

**Published:** 2025-09-01

**Authors:** Anna Paula Ferrari, Juliane Andrade, Cristina Maria Garcia de Lima Parada

**Affiliations:** I Universidade Estadual Paulista “Júlio de Mesquita Filho”. Botucatu, São Paulo, Brazil

**Keywords:** Teaching, Teaching Methods, Active Learning, Sustainable Development Goals, Nursing, Enseñanza, Métodos de Enseñanza, Aprendizaje Activo, Metas de Desarrollo Sostenible, Enfermería

## Abstract

**Objectives:**

to report the experience of including the topics of planetary health and Sustainable Development Goals in undergraduate nursing education.

**Methods:**

this is an experience report, in the context of an integrated curriculum, based on professional competence and that explores active teaching-learning methodologies in an undergraduate nursing course.

**Results:**

the inclusion of the aforementioned topics constitutes an important step towards comprehensive nursing education aligned with current global challenges, transcending the traditional scope and recognizing the vital interconnection between human, animal, and environmental health and planet sustainability.

**Final Considerations:**

reflection was made on the inclusion of planetary health and Sustainable Development Goals in undergraduate nursing education, with the aim of contributing to Higher Education Institutions in general and, specifically, in nursing, mobilizing themselves to address these important topics both in terms of teaching and research.

## RESUMEN

**Objetivos:**

relatar la experiencia de inclusión de la salud planetaria y los Objetivos de Desarrollo Sostenible en la formación de pregrado en enfermería.

**Métodos:**

se trata de un relato de experiencia, en el contexto de un currículo integrado, basado en la competencia profesional y que explora metodologías activas de enseñanza-aprendizaje en una carrera de graduación en enfermería.

**Resultados:**

la inclusión de los temas antes mencionados constituye un paso importante hacia una formación integral de enfermería alineada con los desafíos globales actuales, trascendiendo el alcance tradicional y reconociendo la interconexión vital entre la salud humana, animal y ambiental y la sostenibilidad del planeta.

**Consideraciones Finales:**

se reflexionó sobre la inclusión de la salud planetaria y los Objetivos de Desarrollo Sostenible en la formación de pregrado en enfermería, con el objetivo de contribuir a que las Instituciones de Educación Superior en general y, específicamente, en el área de enfermería, se movilicen para abordar estos importantes temas tanto en lo que respecta a la docencia como a la investigación.

## INTRODUCTION

The discussion on planetary health, a focus of interest since the second half of the 2000s, has been gaining more and more momentum, especially because wars and climate, economic and health crises have threatened life on Earth and brought feelings of uncertainty around the world^(1)^. In that year, 189 countries approved the Millennium Declaration, an agreement made with the aim of making the world safer, more prosperous and fairer. Eight objectives were then established to be achieved by 2015, such as the Millennium Development Goals, including ensuring environmental sustainability, whose focus was directed towards the integration between the principles of sustainable development and government policies and programs^(2)^.

In 2015, in an attempt to minimize the negative impacts of human action on planet health, among other challenges, the United Nations proposed the 2030 Agenda. This is a global plan developed with the participation of 193 member states, and 17 Sustainable Development Goals (SDGs) were established. Targets were set and, by 2030, the countries involved must achieve them. These include measures to eradicate poverty, protect the environment and climate, and ensure that people can enjoy peace and prosperity in all environments^(3)^.

From this perspective, professional training in nursing for the Brazilian Healthcare system, which aims to be guided by individuals’ and communities’ health needs, respect and guarantee human rights, and promote comprehensive and intersectoral education, needed to be reviewed so that issues related to the SDGs and planetary health, among other aspects, were reinforced. Thus, the current Brazilian National Curricular Guidelines (CNE/CES Resolution 3/01) are undergoing an updating process, as stated in Resolution 573 of January 31, 2018, and incorporate, in its Article 21, environmental education and sustainability as cross-cutting topics to the course, proposing, in Article 5, the use of active teaching-learning methodologies (AMs) so that undergraduate students recognizes themselves as a subject in their training process, in a movement of learning to learn, considering and reflecting on the reality of individuals, families, and the community^(4)^.

By encouraging students to participate in their learning process, AMs promote the development of several skills expected of professionals in this century, such as problem-solving, agility, adaptability, collaboration, critical thinking, curiosity and imagination, leadership through influence, initiative, entrepreneurship, effective oral and written communication as well as access to information for careful analysis^(5)^.

The inclusion of relevant cross-cutting topics, such as planetary health and SDGs, combined with the use of AMs, can contribute to the development of a care model that overcomes biologicism and advances towards comprehensiveness^(6)^. This power, however, is little explored when considering the training of nurses, and, in this context, this experience report is presented.

## OBJECTIVES

To report the experience of including planetary health and SDG topics in undergraduate nursing education.

## METHODS

This is an experience report on the inclusion of planetary health and SDGs in undergraduate nursing education. The text is organized into two parts: the first presents the authors’ experience, professors at a public university, in planning and implementing these topics in an integrated curriculum, based on professional competence and exploring the AMs of teaching and learning; and the second presents experience problematization, discussed based on current national and international literature.

### Planning and implementing topics in the curriculum

In 2023, the undergraduate nursing course at a state university in São Paulo launched a new curriculum, integrated and guided by four areas of professional competence (individual healthcare, collective healthcare, management and administration, and education and research), seeking to build a solid foundation for training nursing professionals prepared not only for technical challenges, but also for addressing emerging global issues. The curricular restructuring process, which began in 2017, had the collaboration of all professors involved in the course, including students, graduate students and nurses from different practice settings, in a clear collective approach, demonstrating the commitment to a more holistic training.

Each area of competence is composed of Curricular Units (CUs) which, in turn, are formatted by a competence matrix, a schematic device that organizes, in an interrelated manner, the key actions, purposes, main educational actions, expected performances, academic semester of implementation, and knowledge, skills and attitudes to be achieved.

In this report, the area of competence of collective healthcare and the CU collective health needs from the territory and from a global perspective stand out, developed in the first semester of the first year based on the following thematic chunks: 1 - Introduction to the field of collective health; 2 - University-service-community interaction; 3 - Conceptions of the health-disease process, conceptual models and health needs; 4 - Use of databases in community health diagnosis; 5 - Conceptual models of the health-disease process with a focus on social determination; 6 - Planetary health, climate change, impact on health and SDGs; 7 - Health, environment and sustainability; and 8 - Interaction between man, environment and the health-disease process.

Based on the above, the last three chunks of this CU, which focuses on collective care, are linked to planetary health and the SDGs, highlighting the curriculum’s comprehensive vision by recognizing the interdependence between planet health and conditions. These are not just study topics, but rather an invitation for all those involved to reassess their patterns of thought and actions, seeking harmonious personal insertion on the planet. The chunks are presented below in more detail as well as a summary of objectives and content covered (Chart 1).

**Chart 1:** Objectives and content covered in chunks that comprised the topics of planetary health and Sustainable Development Goals, 2024

Chunk	Objectives	Content
1 - Planetary health, climate change, health impact and SDGs	Relate the impacts of the environment and climate change on human health; Recognize the importance of planetary health and SDGs.	Impacts of the environment on human health: environmental and planetary health; Climate change and health impacts; The SDGs and environmental education in health.
2 - Health, environment and sustainability	Classify sustainable hospitals and healthcare services; Recognize the different types of solid waste and how to proceed with each one, from collection to final destination (segregation and disposal); Identify relevant aspects about waste generation to be considered after purchasing products to be used in healthcare services.	Concepts and objectives of sustainable hospitals and healthcare services; Types of solid waste and their processing; Purchase of products, waste generation, segregation and disposal.
3 - Interaction between man, environment and the health-disease process	Identify environmental situations that influence the community’s health-disease process.	Socio-environmental determinants in the health-disease process.

Chunk 1 (planetary health, climate change, impact on health and SDGs) had the constructivist spiral (CS) as its teaching strategy^(7)^. This demonstrates the institutional commitment to cultivating creative skills and attitudes in students and the pursuit of constructing their own knowledge, rather than learning based on the transmission of information. It was developed in three small groups, with ten students and a facilitator in each. The trigger used was a fictitious problem situation, constructed by the discipline professors and validated by a specialist in teaching AMs.

The intention of this strategy was for students to recognize the importance of planetary health and the SDGs and relate the impacts of the environment and climate change on health.

Chunk 2 (health, environment and sustainability) also used CS in small groups^(7)^, but had two true reports as triggers: one about waste improperly disposed of in landfills and dumps; and another about a report by collectors regarding the incorrect disposal of hospital material. Moreover, technical visits were made to the environmental agents’ cooperative, where waste segregation and recycling are carried out in a medium-sized city in São Paulo, and to the sanitation center of a public university hospital, which highlights the value of applying knowledge in real-world contexts, transcending the classroom.

In this case, the intention was to encourage students to: reflect on the importance of implementing actions to reduce the impacts of health and nursing practices on the environment; recognize the different types of solid waste and their processing, from collection to final destination (segregation and disposal); classify sustainable hospitals and healthcare services; identify relevant aspects about waste generation that need to be considered after purchasing products to be used in healthcare services and how to develop sustainable attitudes in different academic opportunities.

In chunk 3 (human-environment interaction and health-disease process), different teaching strategies were used: educational trip^(8)^, with the showing of a shortened version of the film *The Beginning of Life*, which was intended to provoke feelings and reflections regarding the connection with nature in early childhood and its repercussions in adult life; team-based learning^(9)^, for a theoretical approach to essential concepts; visit to the Environmental School (ES) in the city where the university is located; and subsequent elaboration and sharing of a narrative focused on the experience, in addition to conversation with a specialist, to discuss any doubts or deepen topics related to the chunk, in order to enrich students’ education.

It is important to note that ES is a municipal school that aims, using a specific methodology, to raise awareness of the connection with nature, its particularities and biodiversity. During the visit, different ES professionals interacted with students, contributing to understanding the interrelationship between the environment and well-being as well as raising awareness regarding planet care.

In this chunk, the intention was for students to be able to identify environmental situations that influence the health-disease process of individuals and the community, in addition to perceiving the connection with nature as a prescriptive activity for responsible and sustainable care.

The assessment process for the three chunks described was comprehensive and consisted of two attitudinal assessments and one cognitive assessment, demonstrating genuine concern for students’ comprehensive development. Attitudinal assessments were carried out using an instrument developed by the curriculum implementation management group, considering the different teaching strategies used, aiming to assess students’ performance in learning how to learn. For cognitive assessment, a written test was chosen, prepared in a format applied to reality, and was used as a trigger for questions about the VI Civil Society Report on the 2030 Agenda for Sustainable Development in Brazil, released in 2022, once again bringing up the discussion of this contemporary challenge to nursing education. In this way, facilitators were able to identify the knowledge, skills, and attitudes developed during the semester, aligned with the intentions of each chunk.

Finally, at the end of the semester, one week was set aside for intra-unit activities, aiming to integrate the content of each CU, and another two weeks for inter-unit activities, aimed at integrating the different CUs of that semester. Thus, during the intra-unit week, we sought to integrate all the CU content through conversations with experts in planetary health, and during the inter-unit week, we proposed an activity to observe the campus spaces. This observation was based on a script prepared by the students themselves, after participating in a discussion with a member of the Local Accessibility and Inclusion Committee. It consisted of addressing aspects related to accessibility, safety and the environment, integrating the unit being reported on with the other three developed during the semester: the teaching-learning process of the nurse; the health needs of the individual; and nursing as a healthcare profession and practice of care.

### Problematization of the lived experience

The inclusion of topics related to planetary health and SDGs in undergraduate nursing education represents an important step towards a more comprehensive education aligned with current global challenges. This approach transcends the traditional scope of nursing education, recognizing the vital interconnection between health, the environment and planet sustainability.

Students are challenged to recognize how environmental factors impact the health of individuals and communities, to reflect on the implications of human actions on the environment, and to take a proactive stance in the search for sustainable solutions in order to contribute to preserving the environment, the well-being of future generations, and the construction of a more equitable and healthier world. By addressing issues of global relevance, such as climate change and waste management, and integrating the SDGs into the curriculum aimed at training nurses, the intention to train professionals committed not only to treating diseases, but to promoting health in its broadest sense is evident.

In this context, it can be stated that the curriculum under development is advancing by promoting relevant discussions on planetary health and the SDGs. Both national^(10)^ and international^(11)^ literature highlight the urgency of nursing being appropriate for the interlocution of its practice with the SDGs. Regarding planetary health, scientific production is still scarce, not only when considering research reports, but also reflections and editorials. Thus, there is a gap in publications, especially on the inclusion of this topic in Higher Education Institutions, although environmental health may be present in pedagogical projects of nursing courses in some Brazilian institutions.

It was possible to find an article that reports the experience of a federal university when working on the topic using AMs. In this case, the focus was on promoting reflection on ecosystem protection in the care of individuals and the community^(10)^. As learned from the experience reported here, the authors point out that addressing planetary health in health education transcends the perception of health and disease as isolated aspects, and makes us rethink the context, in its most comprehensive form, involved in this process. They consider this transcendence to be necessary in all segments, especially in sectors promoting training and education, and recognize as a priority the interconnection between human, animal, environmental and, consequently, ecosystem health.

Considering the current enormous global environmental crisis, educational institutions have been reflecting on the importance of addressing this issue in education, exploring its transdisciplinarity, but its effective inclusion in curricula is still incipient. Thus, the curricular experience reported here should be valued, since it advances in addressing planetary health and the SDGs in an integrated manner with the discussion of social determination, structural inequalities, and intersectionalities. As highlighted in the guide to planetary health education (A Framework to Guide Planetary Health Education)^(12)^, future professionals have the opportunity to redirect the trajectory of institutions of various natures, such as health, education, and politics, which reproduce inequalities and, consequently, have repercussions on the planet’s living conditions.

Globally, there is a group of researchers in planetary health who still face several challenges in implementing actions for human sustainability and, consequently, transforming the Anthropocene. The complexity of the issue demands several actions to overcome the challenges that arise, one of which is the inclusion of the topic in the curricula of Higher Education Institutions, using methodologies that provide a critical and transformative view of reality. In this sense, the power of nursing training stands out, given the breadth of their performance and representation. Therefore, this reflection constitutes one of these actions.

In this context, the importance of the topic for professional training and the scarcity of studies that address the Brazilian situation are highlighted. The possibility of reflecting on the current national scenario, where intense environmental challenges are faced, reinforces the need to prepare nurses who can act effectively and ethically in a world increasingly affected by environmental crises. Therefore, incorporating planetary health into nursing education is urgent, as it contributes to training professionals capable of facing the complex challenges of contemporary Brazil and contributing to a healthier and more sustainable future.

It is essential to clarify that the process of putting this experience into practice faced obstacles that could be overcome, the main one being the difficulty that students had in accepting the use of AMs, as proposed, since they were unanimously graduates of a training model that aimed to transmit knowledge traditionally. The facilitators, authors of this article, remained firm with this innovative proposal, especially in the initial moments, which were the most critical. Throughout the process, however, there was understanding and involvement of students with the aforementioned methodologies, at the same time that they realized the relevance of the studied topic.

Finally, the future challenge is to maintain planetary health and the SDGs in transdisciplinary manner in the CUs of the next years of the course. To this end, the professors involved in this report have the important role of disseminating this experience and its positive results, in order to make other professors and students engage with this proposal.

## FINAL CONSIDERATIONS

This text reports on the experience of working on the topics of planetary health and SDGs in undergraduate nursing education using AMs, a problem with an incipient approach in national and international scientific literature. It is believed that, based on this reflection, Higher Education Institutions in general and, specifically, in nursing, can be mobilized, both to look at teaching in the context of planetary health and SDGs and to develop research that involves these topics in teaching and their influence on health practice.
