# Supplementary material for: Inclusion of planetary health and Sustainable Development Goals in nursing training
Source: Rev Bras Enferm. 2025 Sep 1;78(Suppl 1):e20240014. doi: 10.1590/0034-7167-2024-0014 (PMC12407869; doi:10.1590/0034-7167-2024-0014)
Supplement: Translation

# Translation: Inserção da saúde planetária e Objetivos de Desenvolvimento Sustentável na formação do enfermeiro

**DOI:** 10.1590/0034-7167-2024-0014pt

**Authors:** Anna Paula Ferrari, Juliane Andrade, Cristina Maria Garcia de Lima Parada

**Affiliations:** I Universidade Estadual Paulista “Júlio de Mesquita Filho”. Botucatu, São Paulo, Brasil

**Keywords:** Ensino, Métodos de Ensino, Aprendizagem Ativa, Objetivos de Desenvolvimento Sustentável, Enfermagem

## Abstract

**Objetivos:**

relatar a experiência da inserção das temáticas saúde planetária e Objetivos de Desenvolvimento Sustentável no ensino de graduação em enfermagem.

**Métodos:**

trata-se de relato de experiência, no contexto de um currículo integrado, baseado em competência profissional e que explora as metodologias ativas de ensino-aprendizagem em curso de graduação em enfermagem.

**Resultados:**

a inserção dos temas citados constitui passo importante na direção da formação em enfermagem abrangente e alinhada aos desafios globais atuais, transcendendo ao escopo tradicional e reconhecendo a interconexão vital entre a saúde humana, animal, ambiental e a sustentabilidade do planeta.

**Considerações Finais:**

refletiu-se sobre a inserção da saúde planetária e Objetivos de Desenvolvimento Sustentável no ensino de graduação de enfermagem, com a finalidade de contribuir para que Instituições de Ensino Superior em geral e, especificamente, na área da enfermagem, mobilizem-se para abordagem dessas importantes temáticas tanto no que diz respeito ao ensino quanto à pesquisa.

## INTRODUÇÃO

A discussão sobre saúde planetária, alvo de interesse desde a segunda metade dos anos 2000, vem ganhado cada vez mais força, especialmente porque guerras e crises climática, econômica e sanitária têm ameaçado a vida na terra e acarretado sensações de incertezas ao redor do mundo^(1)^. No referido ano, 189 países aprovaram a Declaração do Milênio, acordo realizado com o intuito de tornar o mundo mais seguro, próspero e justo. Foram estabelecidos, então, oito objetivos a serem alcançados até 2015, como os Objetivos de Desenvolvimento do Milênio, incluindo a garantia da sustentabilidade ambiental, cujo foco foi direcionado para a integração entre os princípios do desenvolvimento sustentável e as políticas e programas de governo^(2)^.

A partir de 2015, ainda na tentativa de minimizar os impactos negativos da ação humana na saúde do planeta, entre outros desafios, foi proposta a Agenda 2030 da Organização das Nações Unidas. Trata-se de plano global cuja elaboração contou com a participação de 193 estados membros, e foram estabelecidos 17 Objetivos de Desenvolvimento Sustentável (ODS). Metas foram elencadas e, até 2030, os países envolvidos precisam atingi-las. Essas envolvem medidas para erradicação da pobreza, proteção do meio ambiente e clima, e garantia de que as pessoas possam desfrutar de paz e prosperidade em todos os ambientes^(3)^.

Nessa perspectiva, a formação profissional em enfermagem para o Sistema Único de Saúde, que visa pautar-se nas necessidades de saúde das pessoas e comunidades, respeitar e garantir os direitos humanos, além de promover a educação integral e intersetorial, precisou ser revista, para que questões relacionadas aos ODS e saúde planetária, entre outros aspectos, fossem reforçadas. Assim, as Diretrizes Curriculares Nacionais vigentes (Resolução CNE/CES nº 3/ 01) passam por processo de atualização, como consta da Resolução nº 573, de 31 de janeiro de 2018, e incorpora, em seu Artigo 21º, a educação ambiental e sustentabilidade como temas transversais ao curso, propondo, no Artigo 5º, a utilização das metodologias ativas (MAs) de ensino-aprendizagem, para que o graduando reconheça-se como sujeito no seu processo de formação, em um movimento de aprender a aprender, considerando e refletindo sobre a realidade dos indivíduos, famílias e comunidade^(4)^.

Ao estimular a participação dos estudantes no seu processo de aprendizado, as MAs promovem o desenvolvimento de diversas competências esperadas do profissional deste século, como solução de problemas, agilidade, adaptabilidade, colaboração, pensar criticamente, curiosidade e imaginação, liderança por influência, iniciativa, empreendedorismo, comunicação oral e escrita eficazes, além de acesso à informação para análise criteriosa^(5)^.

A inserção de temas transversais relevantes, como saúde planetária e ODS, aliada ao uso de MAs, pode contribuir com o desenvolvimento de um modelo de cuidado que supere o biologicismo e avance no sentido da integralidade^(6)^. Essa potência, porém, é pouco explorada quando se considera a formação de enfermeiros, e, neste contexto, apresenta-se este relato de experiência.

## OBJETIVOS

Relatar a experiência da inserção das temáticas de saúde planetária e ODS no ensino de graduação em enfermagem.

## MÉTODOS

Trata-se relato de experiência sobre a inserção das temáticas saúde planetária e ODS no ensino de graduação em enfermagem. O texto está organizado em duas partes: na primeira, apresenta-se a vivência das autoras, docentes de universidade pública, no planejamento e implementação desses temas em currículo integrado, baseado em competência profissional e que explora as MAs de ensino-aprendizagem; e na segunda, apresenta-se a problematização sobre a experiência vivenciada, discutida a partir da literatura nacional e internacional vigente.

### Planejamento e implementação dos temas no currículo

Em 2023, o curso de graduação em enfermagem de universidade estadual paulista iniciou novo currículo, integrado e orientado por quatro áreas de competência profissional (cuidado individual em saúde, cuidado coletivo em saúde, gestão e gerenciamento, e educação e pesquisa), buscando construir sólido alicerce para a formação de profissionais de enfermagem preparados não apenas para desafios técnicos, mas também para a abordagem de questões globais emergentes. O processo de reestruturação curricular, que teve início em 2017, contou com a colaboração de todos os professores envolvidos no curso, como discentes, egressos e enfermeiros de diferentes cenários de prática, em clara abordagem coletiva, evidenciando o compromisso com uma formação mais holística.

Cada área de competência é composta por Unidades Curriculares (UCs) que, por sua vez, são formatadas por uma matriz de competência, dispositivo esquemático que organiza, de forma inter-relacionada, as ações-chave, propósitos, principais ações educacionais, desempenhos esperados, semestre letivo de implementação, e conhecimentos, habilidades e atitudes a serem alcançados.

No presente relato, destacam-se a área de competência do cuidado coletivo em saúde e a UC necessidades de saúde do coletivo a partir do território e em perspectiva global, desenvolvida no primeiro semestre do primeiro ano a partir dos seguintes blocos temáticos: 1 - Introdução ao campo de saúde coletiva; 2 - Interação universidade-serviço-comunidade; 3 - Concepções do processo de saúde-doença, modelos conceituais e necessidades de saúde; 4 - Uso das bases de dados no diagnóstico de saúde da comunidade; 5 - Modelos conceituais do processo de saúde-doença com enfoque na determinação social; 6 - Saúde planetária, mudanças climáticas, impacto na saúde e ODS; 7 - Saúde, meio ambiente e sustentabilidade; e 8 - Interação homem, meio ambiente e processo de saúde-doença.

Pelo exposto, os três últimos blocos desta UC, que é voltada ao cuidado coletivo, estão ligados à saúde planetária e aos ODS, evidenciando a visão abrangente do currículo, ao reconhecer a interdependência entre saúde e condições do planeta. Não se trata apenas de tópicos de estudo, mas sim constitui convite para que todos os envolvidos reavaliem seus padrões de pensamento e ações, buscando a inserção pessoal harmônica no planeta. A seguir, são apresentados os blocos com mais detalhes, bem como síntese relativa aos objetivos e conteúdos abordados (Quadro 1).

**Quadro 1:** Objetivos e conteúdos abordados nos blocos que compuseram as temáticas saúde planetária e Objetivos de Desenvolvimento Sustentável, 2024

Bloco	Objetivos	Conteúdo
1 - Saúde planetária, mudanças climáticas, impacto na saúde e ODS	Relacionar os impactos do meio ambiente e das mudanças climáticas na saúde humana; Reconhecer a importância da saúde planetária e ODS.	Impactos do meio ambiente na saúde humana: saúde ambiental e planetária; Mudanças climáticas e impactos na saúde; Os ODS e educação ambiental em saúde.
2 - Saúde, meio ambiente e sustentabilidade	Classificar hospitais e serviços de saúde sustentáveis; Reconhecer os diversos tipos de resíduos sólidos e como deve se proceder com cada um, desde a coleta até o destino final (segregação e descarte); Identificar aspectos relevantes sobre a geração de resíduos a serem considerados após a compra de produtos a serem usados em serviços de saúde.	Conceitos e objetivos de hospitais e serviços de saúde sustentáveis; Tipos de resíduos sólidos e seu processamento; Compra de produtos, geração de resíduos, segregação e descarte.
3 - Interação homem, meio ambiente e processo de saúde-doença	Identificar situações ambientais que influenciam o processo de saúde-doença da comunidade.	Determinantes socioambientais no processo de saúde-doença.

O bloco 1 (saúde planetária, mudanças climáticas, impacto na saúde e ODS) teve como estratégia de ensino a espiral construtivista (EC)^(7)^. Assim, evidencia-se o compromisso institucional de cultivar nos estudantes habilidades e atitudes criativas e de busca da construção do próprio conhecimento, em detrimento do aprendizado baseado na transmissão de informações. Foi desenvolvida em três pequenos grupos, com dez alunos e um facilitador em cada. O disparador utilizado foi uma situação problema fictícia, construída pelas docentes da disciplina e validada por especialista em MAs de ensino.

A intencionalidade desta estratégia foi que os estudantes reconhecessem a importância da saúde planetária e dos ODS e relacionassem os impactos do meio ambiente e das mudanças climáticas na saúde.

O bloco 2 (saúde, meio ambiente e sustentabilidade) também utilizou a EC em pequenos grupos^(7)^, mas teve duas reportagens verídicas como disparadores: uma sobre lixo mal descartado em aterros e lixões; e outra sobre a denúncia de catadores em relação ao descarte incorreto de material hospitalar. De forma complementar, foram realizadas visitas técnicas à cooperativa de agentes ambientais, local onde são realizadas a segregação e reciclagem de resíduos de município paulista de médio porte, e ao núcleo de higienização de um hospital universitário público, o que evidencia a valorização da aplicação do conhecimento em contextos do mundo real, transcendendo a sala de aula.

Nesse caso, a intencionalidade foi estimular os estudantes a: refletirem sobre a importância da implementação de ações para reduzir os impactos das práticas de saúde e enfermagem ao meio ambiente; reconhecerem os diversos tipos de resíduos sólidos e seu processamento, desde a coleta até o destino final (segregação e descarte); classificarem hospitais e serviços de saúde sustentáveis; identificarem aspectos relevantes sobre a geração de resíduos e que precisam ser considerados após a compra de produtos a serem usados em serviços de saúde e como desenvolver atitudes sustentáveis em diferentes oportunidades acadêmicas.

No bloco 3 (interação homem, meio ambiente e processo de saúde-doença), diferentes estratégias de ensino foram utilizadas: viagem educacional^(8)^, com a exposição da versão reduzida do filme *O começo da vida*, que teve a intencionalidade de provocar sentimentos e reflexões em relação à conexão com a natureza na primeira infância e repercussões na vida adulta; aprendizagem baseada em equipes^(9)^, para abordagem teórica de conceitos essenciais; visita à Escola do Meio Ambiente (EMA) do município onde a universidade está inserida; e posterior elaboração e compartilhamento de narrativa focada na vivência, além de conversa com especialista, para discussão de eventuais dúvidas ou aprofundamento de temas relativos ao bloco, de forma a enriquecer a formação dos estudantes.

Destaca-se que a EMA é uma escola municipal que visa, a partir de metodologia específica, sensibilizar para a percepção de conexão com a natureza, suas particularidades e biodiversidade. Durante a visita, diferentes profissionais da EMA interagiram com os estudantes, contribuindo para o entendimento da inter-relação entre meio ambiente e bem-estar, bem como para a sensibilização com relação ao cuidado do planeta.

Neste bloco, a intencionalidade foi que os estudantes conseguissem identificar situações ambientais que influenciam o processo de saúde-doença do indivíduo e da comunidade, além de perceber a conexão com a natureza como atividade prescritiva ao cuidado responsável e sustentável.

O processo avaliativo dos três blocos descritos foi abrangente e composto por duas avaliações atitudinais e uma avaliação cognitiva, demonstrando a preocupação genuína com o desenvolvimento integral dos estudantes. As avaliações atitudinais foram realizadas a partir de instrumento elaborado pelo grupo gestor de implantação do currículo, considerando as diferentes estratégias de ensino utilizadas, visando avaliar o desempenho do aluno no sentido de aprender a aprender. Para a avaliação cognitiva, optou-se pela prova escrita, elaborada em formato aplicado à realidade, sendo utilizado como disparador para as questões sobre o VI Relatório Luz da Sociedade Civil da Agenda 2030 de Desenvolvimento Sustentável Brasil, divulgado em 2022, trazendo mais uma vez a discussão deste desafio contemporâneo à formação em enfermagem. Dessa forma, as facilitadoras puderam identificar o conhecimento, habilidade e atitude desenvolvidos durante o semestre, alinhados às intencionalidades de cada bloco.

Por fim, ao final do semestre, foi destinada uma semana para atividades intraunidades, visando integrar o conteúdo de cada UC e outras duas semanas para atividades interunidades, voltadas à integração das diferentes UCs daquele semestre. Dessa forma, na semana intraunidade, buscou-se integrar todo conteúdo da UC a partir de conversas com especialistas da área da saúde planetária, e na semana interunidades, propôs-se atividade de observação dos espaços do *campus*. Tal observação teve como base roteiro elaborado pelos próprios alunos, após participarem de discussão com um membro da Comissão Local de Acessibilidade e Inclusão. Consistiu na abordagem de aspectos relacionados à acessibilidade, segurança e meio ambiente, integrando a unidade que está sendo relatada às outras três desenvolvidas no semestre: processo de ensino-aprendizagem do enfermeiro; necessidades de saúde do indivíduo; e enfermagem como profissão da saúde e prática de cuidar.

### Problematização sobre a experiência vivenciada

A inserção das temáticas relativas à saúde planetária e ODS no ensino de graduação em enfermagem representa passo importante na direção de uma formação mais abrangente e alinhada aos desafios globais atuais. Essa abordagem transcende o escopo tradicional da formação em enfermagem, reconhecendo a interconexão vital entre saúde, ambiente e sustentabilidade do planeta.

Os estudantes são desafiados a reconhecer como os fatores ambientais impactam a saúde das pessoas e comunidades, a refletir sobre as implicações das ações humanas no meio ambiente e a assumir postura proativa na busca por soluções sustentáveis, de forma a contribuir com a preservação do meio ambiente, com o bem-estar das futuras gerações e construção de um mundo mais equitativo e saudável. Ao contemplar questões de relevância global, como mudanças climáticas e gestão de resíduos, e integrar os ODS no currículo voltado à formação do enfermeiro, evidencia-se a intenção de formar profissional comprometido não apenas com o tratamento de doenças, mas com a promoção da saúde em seu sentido mais amplo.

Nesse contexto, pode-se afirmar que o currículo em desenvolvimento avança ao promover discussões relevantes sobre saúde planetária e ODS. Tanto a literatura nacional^(10)^ quanto a internacional^(11)^ trazem a urgência da enfermagem estar apropriada da interlocução da sua prática com os ODS. Quanto à saúde planetária, a produção científica ainda é escassa, não só quando se consideram relatos de pesquisas, mas também reflexões e editoriais. Dessa forma, percebe-se um hiato de publicações, especialmente sobre a inclusão dessa temática nas Instituições de Ensino Superior, apesar de a saúde ambiental poder estar presente em projetos pedagógicos de cursos de enfermagem de algumas instituições brasileiras.

Foi possível encontrar um artigo que relata a experiência de universidade federal ao trabalhar a temática utilizando MAs. Neste caso, o foco esteve na promoção de reflexão sobre a proteção do ecossistema no cuidado do indivíduo e da coletividade^(10)^. Assim como apreendido da experiência aqui relatada, os autores apontam que abordar a saúde planetária no ensino em saúde transcende a percepção de saúde e doença como aspectos isolados, e faz repensar o contexto, em sua forma mais abrangente, envolvido nesse processo. Consideram ser essa transcendência necessária em todos os seguimentos, em especial nos setores promotores da formação e educação, e reconhecem como prioritária a interconexão entre a saúde humana, animal, ambiental e, consequentemente, dos ecossistemas.

Diante da enorme crise ambiental planetária vivenciada na atualidade, reflexões vêm acontecendo em instituições de ensino sobre a importância da abordagem desta temática na educação, explorando sua transdisciplinaridade, mas a inserção efetiva nos currículos é ainda incipiente. Dessa maneira, a experiência curricular aqui relatada deve ser valorizada, visto que avança ao abordar a saúde planetária e ODS de forma integrada à discussão da determinação social, das desigualdades estruturais e interseccionalidades. Conforme salientado no Guia sobre Saúde Planetária (*A framework to guide planetary health education*)^(12)^, futuros profissionais têm possibilidade de redirecionar o trajeto de instituições de diversas naturezas, como da saúde, educação e política, que reproduzem as desigualdades e, consequentemente, repercutem nas condições de vida do planeta.

Globalmente, há um grupo de pesquisadores no campo da saúde planetária que ainda enfrenta diversos desafios, no sentido de implementar ações de sustentabilidade humana e, consequentemente, da transformação do antropoceno. A complexidade da questão demanda diversas ações para vencer os desafios que se impõem, sendo uma delas a inclusão da temática nos currículos de Instituições de Ensino Superior, com o uso de metodologias que proporcionem olhar crítico e transformador da realidade. Nesse sentido, destaca-se a potência da formação do enfermeiro, visto a amplitude de sua atuação e representação. Logo, a presente reflexão configura uma dessas ações.

Nesse contexto, destaca-se tanto a importância da temática para a formação profissional quanto a escassez de estudos que abordem a situação brasileira. A possibilidade de refletir sobre o cenário nacional atual, onde intensos desafios ambientais são enfrentados, reforça a necessidade de preparar enfermeiros que possam atuar, de forma eficaz e ética, em um mundo cada vez mais afetado por crises ambientais. Portanto, incorporar a saúde planetária na educação em enfermagem é urgente, por contribuir com a formação de profissionais capacitados para enfrentar os complexos desafios do Brasil contemporâneo e contribuir para um futuro mais saudável e sustentável.

É fundamental elucidar que o processo para colocar esta vivência em prática enfrentou obstáculos passíveis de superação, sendo o principal a dificuldade de os estudantes aceitarem o uso de MAs, conforme proposto, uma vez que são egressos, de forma unânime, de modelo de formação que visava transmitir o conhecimento de forma tradicional. As facilitadoras, autoras deste artigo, mantiveram-se firmes com essa proposta inovadora, especialmente nos momentos iniciais, que foram os mais críticos. Ao longo do processo, porém, houve a compreensão e o envolvimento dos estudantes com as citadas metodologias, ao mesmo tempo em que perceberam a relevância da temática estudada.

Por fim, aponta-se como desafio futuro manter a saúde planetária e os ODS de forma transdisciplinar nas UCs dos próximos anos do curso. Para tanto, as docentes envolvidas com o presente relato têm o importante papel de divulgar essa experiência e seus resultados positivos, de forma a fazer com que outros docentes e estudantes se engajem com essa proposta.

## CONSIDERAÇÕES FINAIS

Este texto relata a experiência de trabalhar as temáticas de saúde planetária e ODS no ensino de graduação de enfermagem com o uso de MAs, problemática com abordagem insipiente na literatura científica nacional e internacional. Acredita-se que, a partir desta reflexão, as Instituições de Ensino Superior em geral e, especificamente, na área da enfermagem, possam ser mobilizadas, tanto para olhar o ensino no contexto da saúde planetária e dos ODS quanto para desenvolverem pesquisas que envolvam esses temas no ensino e sua influência na prática em saúde.
